# Supplementary material for: Long-term potentiation and depression regulatory microRNAs were highlighted in Bisphenol A induced learning and memory impairment by microRNA sequencing and bioinformatics analysis
Source: PLoS One. 2023 Jan 19;18(1):e0279029. doi: 10.1371/journal.pone.0279029 (PMC9851566; doi:10.1371/journal.pone.0279029)
Supplement: S1 Table — (DOCX) [file pone.0279029.s001.docx]

**S1 Table. Primers used in real-time PCR.**

| **Gene** | **Sequence of Primer (5'-3')** | |
| --- | --- | --- |
| mmu-miR-24-3p | RT | GTCGTATCCAGTGCGTGTCGTGGAGTCGGCAATTGCACTGGATACGACCTGTTC |
|  | F | TGGCTCAGTTCAGCAGGAA |
|  | R | AGTGCGTGTCGTGGAGTC |
| mmu-miR-182-5p | RT | GTCGTATCCAGTGCGTGTCGTGGAGTCGGCAATTGCACTGGATACGACCGGTGT |
|  | F | TTTGGCAATGGTAGAACTCACAC |
|  | R | AGTGCGTGTCGTGGAGTC |
| mmu-miR-96-5p | RT | GTCGTATCCAGTGCGTGTCGTGGAGTCGGCAATTGCACTGGATACGACAGCAAA |
|  | F | GTGTCGTGGAGTCGGCAATTTGGCACTAGCACATTTTTG |
|  | R | GTGTCGTGGAGTCGGCAA |
| mmu-miR-183-5p | RT | GTCGTATCCAGTGCGTGTCGTGGAGTCGGCAATTGCACTGGATACGACAGTGAA |
|  | F | GTGTCGTGGAGTCGGCAATATGGCACTGGTAGAATTCAC |
|  | R | GTGTCGTGGAGTCGGCAA |
| mmu-miR-193a-3p | RT | GTCGTATCCAGTGCGTGTCGTGGAGTCGGCAATTGCACTGGATACGACACTGGG |
|  | F | AACTGGCCTACAAAGTCCCA |
|  | R | AGTGCGTGTCGTGGAGTC |
| mmu-miR-125a-3p | RT | GTCGTATCCAGTGCGTGTCGTGGAGTCGGCAATTGCACTGGATACGACGGCTCC |
|  | F | ACAGGTGAGGTTCTTGGGA |
|  | R | AGTGCGTGTCGTGGAGTC |
| mmu-miR-10b-3p | RT | GTCGTATCCAGTGCGTGTCGTGGAGTCGGCAATTGCACTGGATACGACTATTCC |
|  | F | GTGTCGTGGAGTCGGCAACAGATTCGATTCTAGGGGA |
|  | R | GTGTCGTGGAGTCGGCAA |
| mmu-miR-10b-5p | RT | GTCGTATCCAGTGCGTGTCGTGGAGTCGGCAATTGCACTGGATACGACCACAAA |
|  | F | TACCCTGTAGAACCGAATTTG |
|  | R | TGCGTGTCGTGGAGTC |

RT: Stem-loop RT primer; F: PCR Forward Primer; R: PCR Reverse primer
