# Supplementary material for: Long-term potentiation and depression regulatory microRNAs were highlighted in Bisphenol A induced learning and memory impairment by microRNA sequencing and bioinformatics analysis
Source: PLoS One. 2023 Jan 19;18(1):e0279029. doi: 10.1371/journal.pone.0279029 (PMC9851566; doi:10.1371/journal.pone.0279029)
Supplement: S2 Table — (DOCX) [file pone.0279029.s002.docx]

**S2 Table. Full name of miRNAs’ target genes.**

| Gria1 | glutamate receptor, ionotropic, [AMPA1 (alpha 1)](https://david.ncifcrf.gov/geneReportFull.jsp?rowids=14799) |
| --- | --- |
| Gria3 | Glutamate receptor, ionotropic, [AMPA3 (alpha 3)](https://david.ncifcrf.gov/geneReportFull.jsp?rowids=14799) |
| Camk2 | Calcium/calmodulin-dependent protein kinase II |
| Camk4 | Calcium/calmodulin-dependent protein kinase IV |
| Gnai1 | [guanine nucleotide binding protein (G protein), alpha inhibiting 1](https://david.ncifcrf.gov/geneReportFull.jsp?rowids=14677) |
| Gnai3 | [guanine nucleotide binding protein (G protein), alpha inhibiting 3](https://david.ncifcrf.gov/geneReportFull.jsp?rowids=14679) |
| Gnaz | [guanine nucleotide binding protein, alpha z subunit](https://david.ncifcrf.gov/geneReportFull.jsp?rowids=14687) |
| Gnaq | [Guanine nucleotide binding protein, alpha q polypeptide](https://david.ncifcrf.gov/geneReportFull.jsp?rowids=14682) |
| Itpr1 | Inositol 1,4,5-triphosphate receptor (type 1) |
| Itpr2 | Inositol 1,4,5-triphosphate receptor (type 2) |
| Map2k1 | mitogen-activated protein kinase kinase 1 |
| Grm1 | [glutamate receptor, metabotropic 1](https://david.ncifcrf.gov/geneReportFull.jsp?rowids=14816) |
| Grm5 | [glutamate receptor, metabotropic 5](https://david.ncifcrf.gov/geneReportFull.jsp?rowids=14816) |
| Grin2b | [glutamate receptor, ionotropic, NMDAR2B](https://david.ncifcrf.gov/geneReportFull.jsp?rowids=14812) |
| PLCβ4 | Phospholipase C, beta 4 |
| Pla2g4e | Phospholipase A2 |
| Prkca | [protein kinase C, alpha](https://david.ncifcrf.gov/geneReportFull.jsp?rowids=18750) |
| Prkcb | [protein kinase C, beta](https://david.ncifcrf.gov/geneReportFull.jsp?rowids=18751) |
| Kras | [Kirsten rat sarcoma viral oncogene homolog](https://david.ncifcrf.gov/geneReportFull.jsp?rowids=16653) |
| Braf | [Braf transforming gene](https://david.ncifcrf.gov/geneReportFull.jsp?rowids=109880) |
| Rap1a | [RAS related protein 1a](https://david.ncifcrf.gov/geneReportFull.jsp?rowids=215449) |
| Rap1b | [RAS related protein 1b](https://david.ncifcrf.gov/geneReportFull.jsp?rowids=215449) |
| Prkacb | [protein kinase, cAMP dependent, catalytic, beta](https://david.ncifcrf.gov/geneReportFull.jsp?rowids=18747) |
| Rps6ka3 | [ribosomal protein S6 kinase polypeptide 3](https://david.ncifcrf.gov/geneReportFull.jsp?rowids=20111) |
