# Supplementary material for: Long-term potentiation and depression regulatory microRNAs were highlighted in Bisphenol A induced learning and memory impairment by microRNA sequencing and bioinformatics analysis
Source: PLoS One. 2023 Jan 19;18(1):e0279029. doi: 10.1371/journal.pone.0279029 (PMC9851566; doi:10.1371/journal.pone.0279029)
Supplement: Attachment — Submitted filename: Response to Reviewers.docx [file pone.0279029.s004.docx]

**The responses to the reviewers’ comments are as following:**

**Review Comments to the Author**
Please use the space provided to explain your answers to the questions above. You may also include additional comments for the author, including concerns about dual publication, research ethics, or publication ethics. (Please upload your review as an attachment if it exceeds 20,000 characters)

**Reviewer #1:** The authors have addressed most of the previous comments. Unfortunately, some issues remain to be clarified.
**Response:** Thank you very much for reviewing our manuscript and giving us so many useful suggestions. Your comments have helped us to improve our article significantly. We highlighted all changes in yellow in our revised manuscript.

**Comment 1**: It is described in the section “Hippocampus tissue collection” of Material and Methods that tissue from coordinate -2 mm AP, ±1.8 mm M/L, and 1.5 DV was collected, which corresponds to a small portion of CA1 (sub pyramidal part of CA1 stratum radiatum). Please describe whether only this portion of CA1 was collected or other regions were also included. If the entire hippocampus were collected, it is not necessary to cite the coordinates. Please, make it clear.
**Response:** Thank you for your helpful suggestion. We are sorry that we did not understand reviewer’s opinion well and wrote the location of hippocampus last time. In our study, the entire hippocampus was collected, because the hippocampus CA1 of mice was too small to collect. We removed the coordinates in this revised manuscript.

**Comment 2：**In the “Western Blot” section, it is not clear how the normalization by GAPDH was performed. Please describe if the GAPDH staining was performed in stripped membranes used to identify the target protein’s antibodies. Also, inform whether the normalization was performed by the ratio from band intensity of target proteins against GAPDH. The blotting membrane provided in the supplementary section seems to be cut and does not contain the entire sample used for quantification (only four bands for each target protein). Besides that, it does not present the standard protein ladder, which aids the identification of the right band to be quantified. Please, it is necessary to disclose the entire membrane with all samples used in the present work.

**Response:** Thank you for your helpful suggestion. In western blot, GAPDH staining was performed in stripped membranes used to identify the target protein’s antibodies, and normalization was performed by the ratio from band intensity of target proteins against GAPDH. We added this information in our revised manuscript.

We are sorry for that we didn’t present the standard protein ladder and disclose the entire membrane before (Our western blot were conducted with old developing equipment which could not merge the protein ladder and gray target bands, therefore we only took the photo of gray target bands, and cut the blotting membrane for saving the antibodies). We really value your suggestion, so we did the western blot again and used new developing equipment from other laboratories this time, and the data were afford in our revised supplement data.

**Comment 3**：In the “Statistical analysis”, it is described that data was submitted to homogeneity and normality test. If all data set follows a normal distribution, please indicate it in the text. If it is not the case, please report the statistical test employed.
**Response:** Thank you for your helpful suggestion. In our study, all data set fellows a normal distribution. We have added this information in our revised manuscript.

**Comment 4**：In figure 2A, please indicate which trial (i.e., trial 1-4) the representative swimming route belongs to and if it was obtained from the same animal.

**Response:** Thank you for your helpful suggestion. The representative swimming routes in figure 2A, we have indicated the representative swimming route of trial 1-4. The representative swimming routes belong to the same animal in each group. We have added this information in our revised manuscript.

**Comment 5：** The miRNA analysis may have been impacted by the low number of samples employed in the analyzed groups, now indicated by the authors. It is possible that the change in miRNA expression after treatment with BPA was affected by the small size sample since some displaced miRNA indicated in the scatter plot were not significant in the statistical test. Please justify the small sample size for the groups.

**Response:** Thank you for your helpful suggestion. As you suggestion, we performed the statistical analysis for the displaced miRNAs indicated in the scatter plot. All the displaced miRNAs were significantly changed, however, the P value varies from 0.042 to <0.001. Therefore, chip or sequencing is the first step to screen differentially expressed miRNAs, Real Time-PCR is used to verify the miRNA expression which screened out. In Table 1 of our revised manuscript, we added the *p*-value of miRNAs with statistical differences in expression between the BPA treatment and control groups.

**Comment 6：**In the result section, please indicate in the text values of the mean and SEM represented in the graphs.

**Response:** Thank you for your helpful suggestion. We have indicated the mean and SEM represented in miRNA analysis in Table 1 of our revised manuscript.

**Minor issues**

**Comment 1:** Line 26-28 Change “Results showed that mice treated with BPA displayed impairments of spatial learning and memory, and the expression of miRNAs in hippocampus changed.” For “Results showed that mice treated with BPA displayed impairments of spatial learning and memory and changes in the expression of miRNAs in the hippocampus.”

**Response:** Thank you for your helpful suggestion. We have corrected this sentence.
**Comment 2:** Line 99- change “BPA induced learning and memory..” for BPA-induced

**Response:** Thank you for your helpful suggestion. We have changed “BPA induced learning and memory..” for BPA-induced.
**Comment 3:** Line 101- idem

**Response:** Thank you for your helpful suggestion. We have added “-”.
**Comment 4:** Line 556-557- idem

**Response:** Thank you for your helpful suggestion. We have added “-”.
**Comment 5:** Line 211 – correct the word “normalrized”

**Response:** Thank you for your helpful suggestion. We have correct the word to “normalized”.
**Comment 6:** Line 213 – Correct the verb tense

**Response:** Thank you for your helpful suggestion. We have correct the verb tense. **Comment 7:** Line 253- Change “4 C for 1h” to “4 °C for 1 h”

**Response:** Thank you for your helpful suggestion. We have correct “4 C for 1h” to “4 °C for 1 h”.

**Comment 8:** Line 271- Please include the antibody’s company full name and the catalog number of the product.

**Response:** Thank you for your helpful suggestion. We have added the antibody’s company full name and the catalog number.

**Comment 9:** In figure 3A, please insert the information in the legend about the blue dots.

**Response:** Thank you for your helpful suggestion. We have insert the information in the legend about the blue dots.

**Comment 10:** Line 427- Change “all of them protein levels..” for “all analyzed protein levels…”

**Response:** Thank you for your helpful suggestion. We have Changed “all of them protein levels..” for “all analyzed protein levels…”

**Comment 11:** Line 447 changes “manipulation” to “involved in”

**Response:** Thank you for your helpful suggestion. We have changed “manipulation” to “involved in”.
